# Heavy metal contamination and environmental risk assessment: a case study of surface water in the Bahr Mouse stream, East Nile Delta, Egypt

**DOI:** 10.1007/s10661-024-12541-1

**Published:** 2024-04-05

**Authors:** Fatma Ramadan, Hamdy E. Nour, Nermin Abdel Wahed, Ahmad Rakha, Abdulgafar K. Amuda, Mohamed Faisal

**Affiliations:** 1 Geology Department, Faculty of Sciences, Zagazig University, Zagazig City, 44519 Egypt; 2 Environmental Affairs, Sharkiya Governorate, Zagazig City, 44511 Egypt; 3 Central Administrations for Environmental Inspection at the Ministry of Environment, Cairo City, 11728 Egypt; 4 Department of Geology, Bayero University Kano, Kano State, 700241 Nigeria; 5 Key Laboratory of Metallogenic Prediction of Nonferrous Metals and Geological Environment Monitoring, Ministry of Education, School of Geosciences and Info-Physics, Central South University, Changsha, 410083 China; 6 Department of Geology, Faculty of Science, Suez Canal University, Ismailia City, 41522 Egypt

**Keywords:** Water Quality Index (WQI), Heavy metals, Surface water pollution, Environmental risk assessment, Sharkiya Governorate, Bahr Mouse stream

## Abstract

Water, as an indispensable constituent of life, serves as the primary source of sustenance for all living things on Earth. The contamination of surface water with heavy metals poses a significant global health risk to humans, animals, and plants. Sharkiya Governorate, situated in the East Nile Delta region of Egypt, is particularly susceptible to surface water pollution due to various industrial, agricultural, and urban activities. The Bahr Mouse Stream, crucial for providing potable water and supporting irrigation activities in Sharkiya Governorate, caters to a population of approximately 7.7 million inhabitants. Unfortunately, this vital water source is exposed to many illegal encroachments that may cause pollution and deteriorate the water resource quality. In a comprehensive study conducted over two consecutive seasons (2019–2020), a total of 38 surface water samples were taken to assess the quantity of heavy metals in surface water destined for human consumption and other applications, supported by indices and statistics. The assessment utilized flame atomic absorption spectrophotometry to determine the concentration of key heavy metals including iron (Fe), manganese (Mn), cadmium (Cd), copper (Cu), lead (Pb), zinc (Zn), nickel (Ni), cobalt (Co), and chromium (Cr). The calculated mean value of the Water Quality Index (WQI) was found to be 39.1 during the winter season and 28.05 during the summer season. This value suggests that the surface water maintains good quality and is suitable for drinking purposes. Furthermore, the analysis indicated that the concentrations of heavy metals in the study area were below the recommended limits set by the World Health Organization and fell within the safe threshold prescribed by Egyptian legislation. Despite the identification of localized instances of illegal activities in certain areas, such as unauthorized discharges, the findings affirm that the Bahr Mouse stream is devoid of heavy metal pollution. This underscores the importance of continued vigilance and regulatory enforcement to preserve the integrity of these vital water resources.

## Introduction

Water is a priceless natural resource that is required for both human survival and the health of ecosystems. Pollution occurs when an aquatic ecosystem is badly damaged by the addition of too many things, rendering the water unfit for its intended usage (Olatunji et al., 2017; Nour et al., 2022a). Among the most pervasive contaminants in aquatic ecosystems are heavy metals, known for their persistence and toxicity, with concentration further exacerbated by biomagnification processes (Dervash & Mushtaq, 2019; Nour & Nouh, 2020). Notably, heavy metals, such as lead, cadmium, and mercury, accumulate in water reservoirs, penetrating the food chain and posing significant ecological risks (Nour, 2015; Pandiyan et al., 2021)‏. Moreover, their consumption has been linked to severe health implications, including cancer and neurological impairment in humans (Alharbi et al., 2023; Al-Kahtany et al., 2023a; Nour et al., 2022b). Anthropogenic activities, such as industrial discharge, domestic sewage, non-point source runoff, and atmospheric precipitation, stand as principal contributors to the infiltration of toxic heavy metals into aquatic systems (Akhtar et al., 2021; Nour et al., 2021; Ramadan et al., 2021). This further exacerbates the global concern over heavy metal contamination in aquatic environments (Al-Kahtany et al., 2023b; Sobhanardakani et al., 2018).

Egypt, a densely populated developing country with a highly arid climate, faces substantial challenges in managing its water resources, given its requirement of 114 BCM annually. With the goals outlined in Egypt Vision 2030 focusing on the preservation of natural water resources, the significance of monitoring heavy metal concentration in surface water becomes paramount amidst challenges posed by climate change, water scarcity, rapid population growth, and infrastructure developments such as the Grand Ethiopian Renaissance Dam construction.

The East Delta region, encompassing Sharkiya, Dakhalia, and Damietta governorates, is characterized by a network of canals, drains, and lakes that traverse and delineate its territory. These water bodies are categorized into freshwater systems, such as the Damietta Branch of the Nile River, and irrigation canals including Baḩr Mouse, El Raiah El Tawfeiki, Bahr Abu Akhder, and Ismailia Canal, as well as saline water bodies comprising the Suez Canal, El Temsah Lake, and El Manzala Lake.

**Fig. 1:**
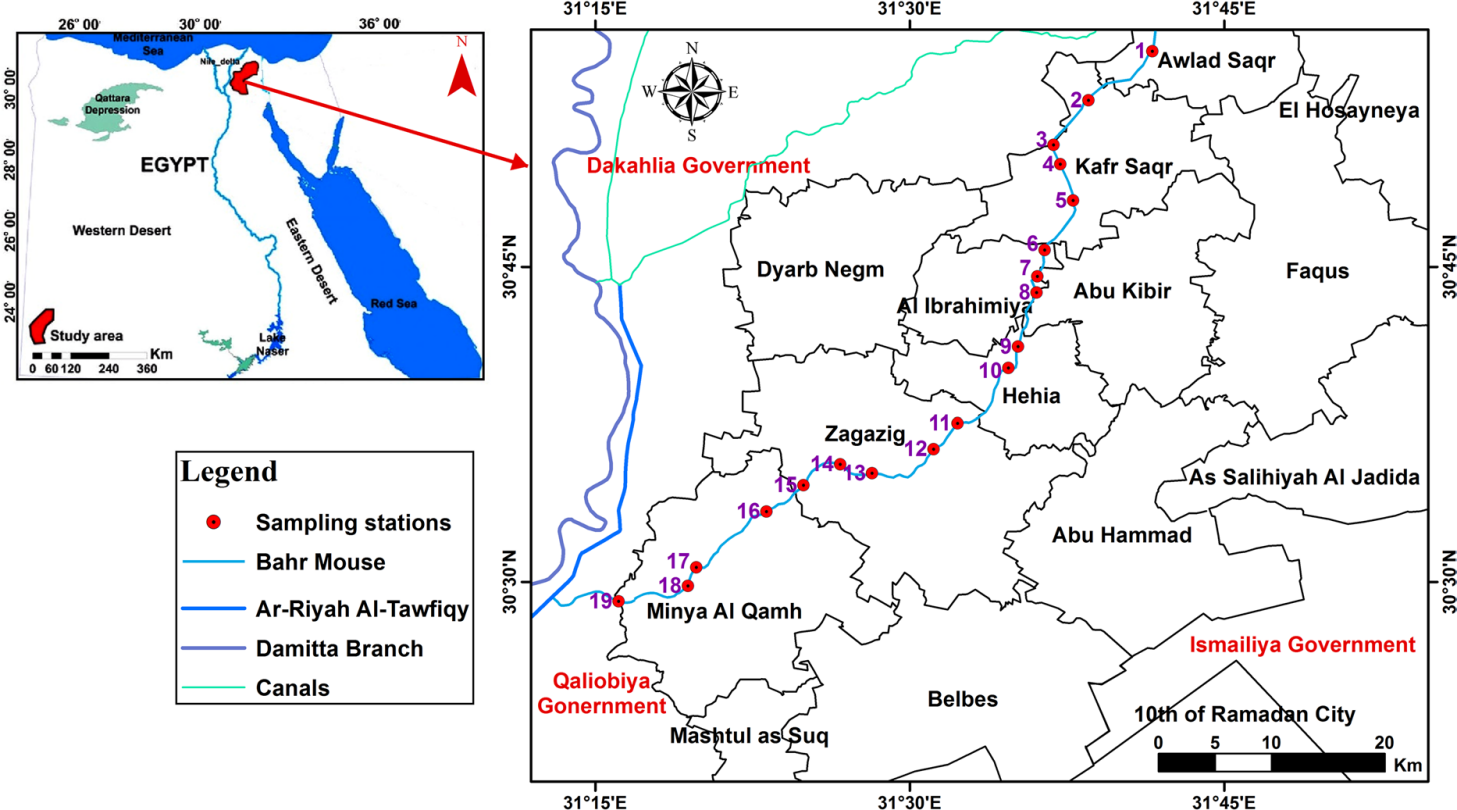
Location map of the Bahr Mouse stream (East Nile Delta, Egypt) showing the stations of water samples

Focusing on the Sharkiya Governorate, our target area covers 4922 km^2^ (1169285 faddan; Fig. 1) and lies within the coordinates of latitude 30° 30′ and 31° 00′ N and longitudinal 31° 15′ and 31° 50′ E, with an elevation of 10 m above the sea level. Notably, the population of Sharkiya governorate is projected to exceed 7.7 million individuals by 2021, as reported by the Central Agency for Public Mobilization and Statistics.

Baḩr Mouse, a crucial freshwater canal originating from the Tawffiky Diversion directly linked to the River Nile near Banha city in the north of Sharkiya Governorate, Egypt (Fig. 1), serves as the main source of drinking water, irrigation, and fishing activities within the region. However, field observations reveal concerning levels of pollution in the Bahr Mouse stream, marked by the presence of various illegal contaminants including garbage, demolition waste, sewage discharge, and industrial effluent from clay brick factories. In addition, it is constantly polluted with amounts of components such as fertilizer residues and human wastes, further deteriorating its quality. Despite the evident pollution burden, systematic research analyzing heavy metal contents and its impact on human health in this area remains scarce. Hence, the primary aim of this study is threefold: (a) to estimate the concentrations of heavy metals in the surface water of the Baḩr Mouse stream, (b) to investigate the physicochemical parameters of the stream water, and (c) to calculate the ecological risk indices of water samples collected from the study area. Through this research, we aim to provide valuable insights into the current state of water quality in the investigated area, shedding light on potential implications for human health and the environment. We emphasize the importance of addressing the adverse effects of recent economic development and rapid population growth on heavy metal levels, urging decision-makers to prioritize measures aimed at safeguarding water resources and public health.

## Materials and methods

### Study region

Baḩr Mouse, a freshwater stream spanning approximately 84 km^2^ with widths ranging from 10 to 20 m, traverses Sharkiya Governorate in Egypt, bounded by latitudes 30° 29′ 16″ N, 31° 12′ 56.8″ N and longitudes 30° 55′ 53.3″ E, 31° 42′ 23.1″ E (Fig. 1). The water depth fluctuates between 1.5 m and a comparatively uncommon 4 m. Notably, Baḩr Mouse meanders through several urban centers including Zagazig, Abu Kibir, Hehia, Al Ibrahimiya, Kafr Saqr, Awlad Saqr, and Minya Al Qamh cities. Surrounding the research area are prominent geographical features: the west is bordered by the Nile River (Damietta Branch), the east by the Suez Canal, the north by Manzala Lake, and the south by the Ismailia Canal (Fig. 1).

The research area’s climate is characterized by a warm winter punctuated by intermittent rainfall, followed by a hot arid summer with moderate humidity, and medium-speed wind. The region experiences an average annual temperature of 20.3 °C, with the highest temperature of 36.7 °C in July and the lowest temperature of 6.4 °C in January. Geological studies by El-Fayoumy (1968) have identified dominant sedimentary formations in the eastern Nile Delta, comprising Tertiary and Quaternary sedimentary sequences. Tertiary rocks include Eocene, Oligocene, Miocene, and Pliocene formations, while Quaternary deposits, predominantly Nile silts (Holocene) interspersed with Pre-Nile deposits, are widely distributed throughout the study area.

### Sample collection and heavy metal analysis

**Fig. 2:**
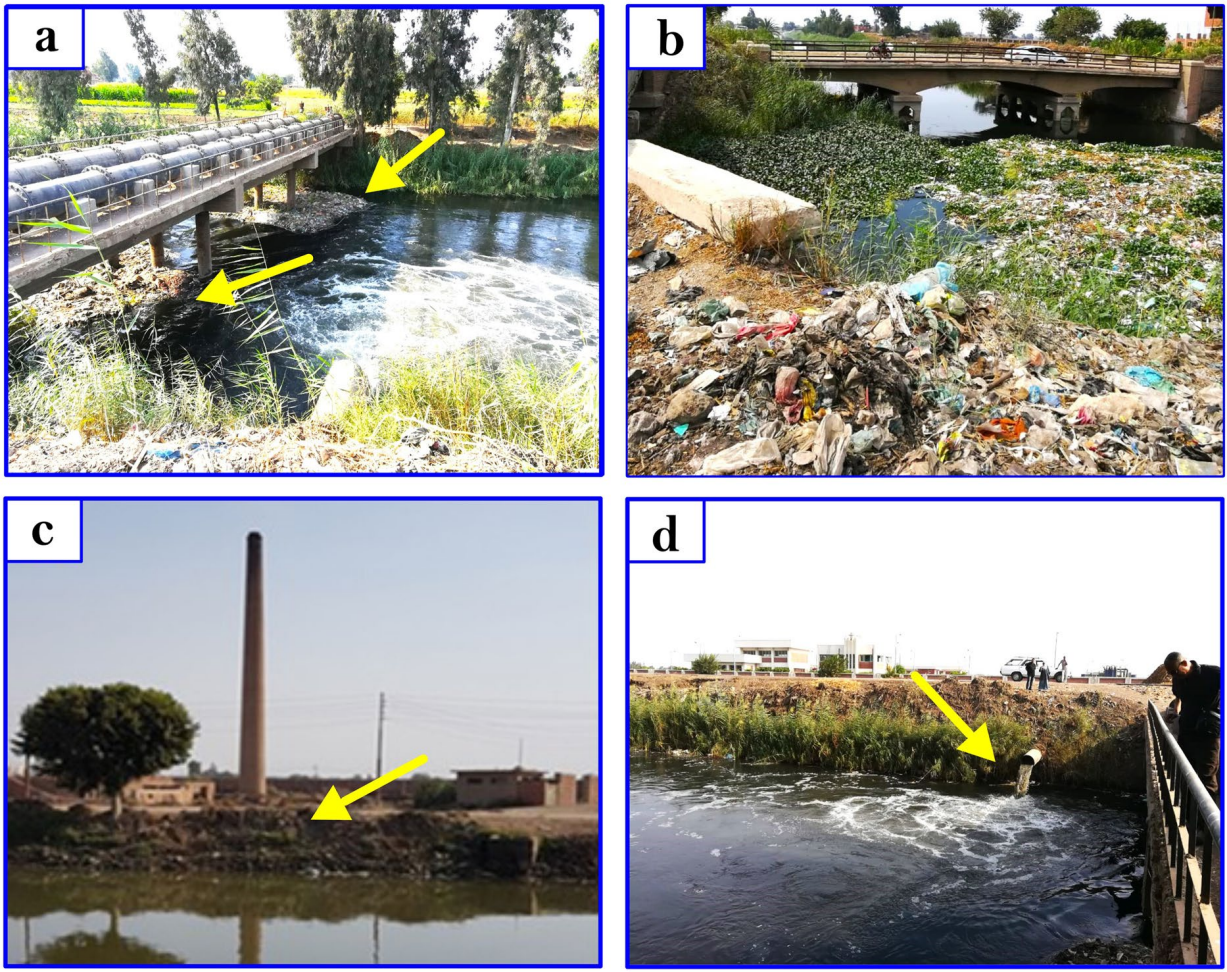
Photographs showing the polution phenomenons in the study area: **a** and **b** the surface and sides of the stream is totally covered with garbage, plastics materials, and dead animals, **c** the waste of materials of Red brick factory on the blank of the stream, **d** the waste water pump of the factory discharge into the stream, the color of water displays highly polluted of the stream

Thirty-eight water samples were taken from nineteen localities in Sharkiya Governorate, covering seasonal periods from December 2018 to August 2019 (Fig. 2). The sampling process involved precise geographical delineation utilizing Geographic Position System (GPS) instruments, and the map of the study area was drawn using Arc GIS (10.1). Each sample, collected from approximately 1 m below the water surface to minimize potential surface contamination, was carefully preserved in sterile 1-l plastic containers. At each location, about 1 l of water was collected. The properties of water samples were measured in situ using a portable calibrated multi-parameter instrument. More specifically, measurements were conducted using a Mercury-in-glass thermometer for temperature (T) and a Horiba multi-parameter water quality checker U-51 for pH, electrical conductivity (EC), and total dissolved solids (TDS) analyses. After in situ measurements, the samples were treated with 1 mm of HNO_3_ in a one-liter sample to arrest microbial activities (APHA, 1995). They were stored in an icebox (at 4 °C,) for transport to the laboratory. Then, 0.45-m membrane filters were used to filter the samples (white rim, Whatman no. 42, Germany). Generally, samples were prepared and analyzed according to the procedures described by Oregioni and Astone (1984) for heavy metals. The Fe, Mn, Cd, Cu, Pb, Zn, Ni, Co, and Cr contents were determined by the flame atomic absorption spectrophotometry (Perkin Elmer Model 2830). The analysis was carried out in the Central Laboratory at the Faculty of Veterinary Medicine, Zagazig University.

Mixed standards were prepared containing Fe, Cu, Zn, Pb, and Cd with concentrations of 0.5 ppm 1.0 ppm 2 ppm, 4 ppm, and 10 ppm. All detection limits are given in micrograms per liter and were determined using elemental standards in dilute aqueous solution. Appropriate drift blank was taken before the analysis of samples. The analytical data quality was guaranteed through the implementation of laboratory quality assurance and quality control methods, including the use of standard operating procedures, calibration with standards, analysis of reagent blanks, recovery of known additions, and analysis of replicates. This is to verify that decontamination procedures and laboratory protocols were adequate (Oregioni and Astone, 1984). Triplicate measurements were made for each sample, with differences between replicates not exceeding 3%, to uphold stringent accuracy standards. The average values of three replicates were taken for each determination. Quantitative estimation of metal concentration was estimated under standardized conditions as described in the instrument manual. Detection limits, established at a 98% confidence level, were determined as follows:1.5 μg/l for Zn, 5 μg/l for Pb, 9 μg/l for Cu, 1.5 μg/l for Mn, and 0.8 μg/l for Cd.

### Water quality indices

Five indices (WQI, HEI, Cd, HMPI, and PI) were applied to evaluate the water quality and pollution status of heavy metals in the surface water of Bahr Mouse stream. Water Quality Index (WQI) is a mathematical concept that calculates the combined effect of each standard parameter on the water’s overall quality (APHA, 2012). It can serve as a sign of water pollution due to natural inputs and anthropogenic activity (Muyen et al., 2016). The Weighted Arithmetic Water Quality Index method (WAWQI) developed by Brown et al. (1972) was used in our study to calculate the WQI of surface water by the following equations:

1 $${\text{WQI}}=\sum_{i=1}^{N}{\text{W}}i \times {\text{Q}}i$$

2 $$Wi=\frac{{\text{w}}i}{\sum {\text{w}}i}$$

3 $$wi=\frac{{\text{K}}}{Si}$$

4 $$K=1/\sum_{i=1}^{N}\frac{1}{Si}$$

5 $$Qi=\left(\frac{{\text{M}}i-{\text{V}}o}{{\text{S}}i-{\text{V}}o}\right)\times 100$$

where *N* is the number of parameters, ${\text{W}}i$ is the relative weight of each parameter, ${\text{Q}}i$ is the water quality rating, w $i$ is the weight of each parameter, ∑w $i$ is the sum of weight of all 10 parameters, $K$ is the proportionality constant, S $i$ is the WHO maximum allowable limits for each parameter in mg/L, M $i$ is the measured concentration for each parameter in each water sample, and ${\text{V}}o$ is the ideal value for each parameter, (0 for all) except pH = 7 (Tripaty & Sahu, 2005). WQI were classified into five categories: excellent, (WQI < 25); good, (26–50); poor, (51–75); very poor, (76–100), and > 100 unfit for drinking water consumption. Additionally, the pollution evaluation index provides a good grading system for water quality (Biswas et al., 2017).

Heavy metal Evaluation Index (HEI) is a method used to assess the overall impact of each parameter on the quality of the water (Saleem et al., 2019). Typically, HEI is utilized to determine the pollution level caused by HMs (Dippong et al., 2020). It is calculated by the equation:

6 $${\text{HEI}}=\sum_{i=1}^{N}\frac{{\text{M}}i}{{\text{MAC}}i}$$

where M $i$ is the measured concentration and MAC $i$ is the maximum allowable concentration for each parameter. Nine parameters (Fe, Mn, Cu, Cd, Pb, Zn, Ni, Co, and Cr) were used in the calculation and water samples can be divided into three groups: low (HEI < 10), medium (10 < HEI < 20), and high contamination (HEI > 20) (Ghaderpoori et al., 2018).

Degree of contamination (Cd) summarizes the combined effects of a number of quality parameters considered harmful to domestic water (Backman et al., 1998). It is calculated by:

7 $$Cd ={\sum }_{i=1}^{N}{\text{Cf}}i$$

8 $${\text{Cf}}i=\frac{{\text{M}}i}{{\text{MAC}}i}-1$$

where *N* is the number of parameters, ${\text{Cf}}i$ is the contamination factor of *i*th component, ${\text{M}}i$ is the analytical value of *i*th component, and ${\text{MAC}}i$ is the maximum permissible concentration of *i*th component. According to Al-Ani et al. (1987), the Cd values were classified as, low (Cd < 1), medium (Cd = 1–3), and high contamination (Cd > 3).

The Heavy Metal Pollution Index (HMPI) considers the combined effect of each heavy metal on the overall water quality (Vasant et al., 2016). Based on the significant parameters affecting water chemistry, HMPI is utilized to assess the impact of both anthropogenic and natural activities (Ghaderpoori et al., 2018). According to Mohan (1996), it is calculated by:

9 $${\text{HMPI}}={\sum }_{i=1}^{N}({\text{W}}i\times \mathrm{ Q}i)/{\sum }_{i=1}^{N}(Wi)$$

10 $$Wi=\frac{{\text{w}}i}{\sum {\text{w}}i}$$

11 $$wi=\frac{{\text{K}}}{Si}$$

12 $$K=1/\sum_{i=1}^{N}\frac{1}{Si}$$

13 $$Qi=\left(\frac{{\text{M}}i-{\text{I}}i}{{\text{S}}i-{\text{I}}i}\right)\times 100$$

where *N* is the number of parameters, ${\text{W}}i$ is the relative weight of each parameter, ${\text{Q}}i$ is the water quality rating, w $i$ is the weight of each parameter, ∑w $i$ is the sum of weight of all 10 parameters, $K$ is the proportionality constant, S $i$ is the WHO maximum allowable limits for each parameter in mg/L, M $i$ is the measured concentration for each parameter, and ${\text{I}}i$ is the ideal value for each parameter in each water sample which were taken as zero for all of metals in this study like some research (Dede, 2016; Reza & Singh, 2010). The critical value for HMPI is 100 (Prasad & Bose, 2001).

Pollution Index (PI) is used to evaluate the degree of heavy metal contamination in water samples (Odukoya & Abimbola, 2010). PI is based on individual metal calculations with the equation:

14 $${\text{PI}}=\sum_{i=1}^{N}\frac{(\frac{{\text{M}}i}{Si})}{Nm}$$

where Mi is the measured concentration for each parameter, S $i$ is the WHO maximum allowable limits and Nm is the number of heavy metals. According to Caerio et al. (2005), PI values were classified into six classes: (1) no effect (PI < 1), (2) slightly affected (1–2), (3) moderately affected (2–3), (4) strongly affected (3–5), and (5) seriously affected (˃5).

## Results and discussion

### Physiochemical parameter analyses

**Fig. 3:**
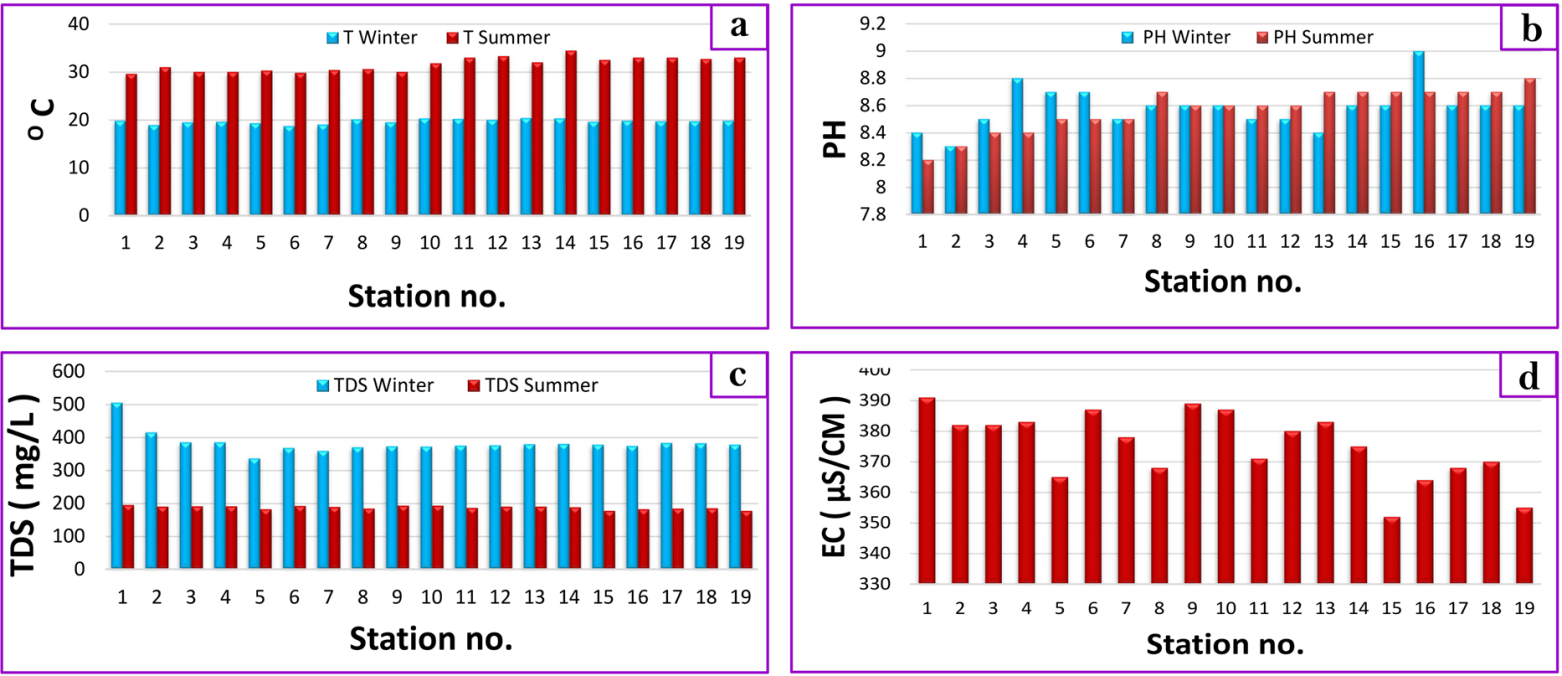
Variation in physiochemical properties of water samples from Bahr Mouse stream (East Nile Delta, Egypt) dueing the period 2018/2019

The properties of the surface water samples were assessed in situ, encompassing water temperature (℃), pH, electrical conductivity (EC) (μs/cm), and total dissolved solids (TDS) (mg/L). Temperature, recognized as a pivotal factor affecting aquatic ecology (Huet, 1986; Ali et al., 2021), exhibits seasonal variations. The measured temperature values were between 18.7–20.4 and 29.6–34.5 °C in winter and summer, respectively (Fig. 3; Table 1).

**Table 1:** Water physicochemical parameters in the Bahr Mouse stream, East Nile Delta, Egypt

Parameter	Winter	Summer	Drinking water standards			Irrigation standards	Aquatic live CCME (2017)
			EWQS (2007)	WHO (2022)	USEPA (2018)	FAO (1994)	
Temperature (°C)	18.7–20.4	29.6–34.5	-	25–30	-	< 35	8–28
pH	8.3–9	8.2–8.8	6.5–8.5	6.5–8.5	6.5–8.5	8.5	6.5–9
TDS (mg/L)	336–506	177–195	1000	500	500	2000	500
EC (μs/cm)	-	352–392	2000	-	-	3000	-

The lowest value (18.7 °C) was recorded at station 6 (Near Izbat Al Bakakrah, Al Ibrahimiya) in winter, while the notable highest recorded temperature (34.5 °C) at station 14 (Tall Hawein, Zagazig) during summer exceeded the permissible limit of WHO (2022) standards. Elevated temperatures speed up chemical reactions which significantly affect the growth and biological activity of aquatic organisms while constraining the diversity of species inhabiting freshwater environments (Magaña, 2020).

Hydrogen ion concentration (pH) regulates the solubility of metals in aquatic ecosystems, with most species thriving within a pH range of 6.0 to 9.0, and exhibiting heightened activity around pH 7 (El-Sayed et al., 2020). pH values of the Bahr Mouse stream varied between 8.3–9 and 8.2–8.8 (alkaline) during winter and summer, respectively (Fig. 3; Table 1). WHO (2022), USEPA (2018), and EWQS (2007) reported that the pH of drinking water should be in the pH range of 6.5–8.5, while the FAO (1994) gives an upper limit of 8.5 for pH in irrigation water. The mean value of 8.5 fell within the permissible range stipulated by international standards. Electrical conductivity (EC) is a sensitive indicator of water contamination, with deviations from normal ranges often indicative of pollution. Naturally, water has a low conductivity, but if liquid effluents are discharged into water bodies, contamination will cause it to rise. The EC of water samples collected during summer ranged from 352 to 391 μs/cm, averaging 375.26 μs/cm. These values fell within the permissible limits of EWQS (2007) and FAO (1994) standards. Total dissolved solids (TDS) concentrations varied between 336–506 and 177–195 mg/L during the winter and summer seasons, respectively (Fig. 3; Table 1). Notably, TDS levels showed a noticeable increase during low water levels in the winter with most values falling below the permissible limit (500 mg/L) set by WHO (2022), USEPA (2018), and EWQS (2007) for drinking water and FAO (1994) for irrigation water.

### Heavy metal analyses

**Fig. 4:**
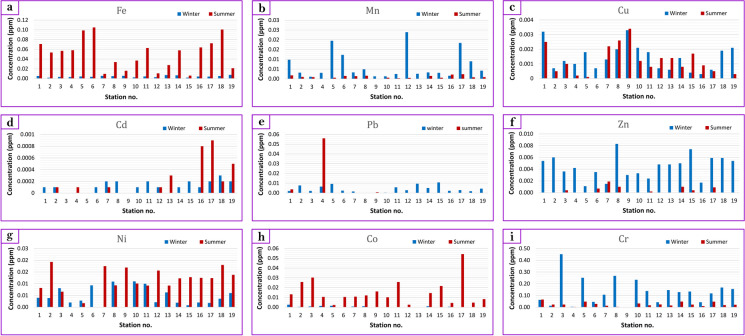
The average distribution of Fe, Mn, Cu, Zn, Pb, Ni, Cd, Co, and Cr contents in the water samples from Bahr Mouse stream (East Nile Delta, Egypt) dueing the period 2018/2019

The seasonal variation in concentrations of water heavy metals is presented in Table 2 and 3, accompanied by a graphical representation in Fig. 4. The hierarchical order of concentrations observed across seasons was as follows: Cr > Fe > Pb > Ni > Co > Mn > Zn > Cu > Cd. Remarkably, the highest recorded iron (Fe) concentration was 0.0074 ppm during winter at station 19 (Minya Al Qamh), whereas it peaked at 0.1049 ppm during the summer at station 6 (Al Ibrahimiya). Conversely, the lowest Fe concentration was noted at 0.0017 ppm during winter at station 2 (Kafr Saqr) and 0.006 ppm during summer at station 15 (Zagazig), with an average concentration of 0.0275 ppm (Fig. 4; Table 2 and 3). The range of Fe concentration (0.0040 to 0.0542 ppm) fell within the safety threshold established by WHO (2022), USEPA (2018), and EWQS (2007) for drinking water, as well as FAO (1994) guidelines for irrigation water. Notably, these concentrations are relatively lower when compared with findings from other local and international studies (Table 4 and 5).

**Table 2:** Water physiochemical properties and heavy metals concentrations (ppm) of water samples of Bahr Mouse in the winter season 2018

Station	Latitude	Longitude	T (°C)	pH	TDS (mg/L)	Fe	Mn	Cu	Cd	Pb	Zn	Ni	Co	Cr
1	N 30° 55′ 16.697″	E 31° 41′ 35.123″	19.8	8.4	506	0.0052	0.0098	0.0032	0.0001	0.002	0.0054	0.004	0.0026	0.0634
2	N 30° 52′ 56.914″	E 31° 38′ 32.474″	18.9	8.3	415	0.0017	0.0032	0.0007	0.0001	0.0077	0.006	0.0039	0.0004	0.0128
3	N 30° 50′ 47.302″	E 31° 36′ 52.077″	19.5	8.5	385	0.0032	0.0011	0.0012	ND	0.0021	0.0036	0.0081	0.0008	0.4518
4	N 30° 49′ 54.102″	E 31° 37′ 11.965″	19.6	8.8	385	0.003	0.0031	0.001	ND	0.0065	0.0042	0.002	0.0014	0.0041
5	N 30° 48′ 9.805″	E 31° 37′ 48.181″	19.3	8.7	336	0.0042	0.0195	0.0018	ND	0.0093	0.0011	0.0028	0.0015	0.2511
6	N 30° 45′ 47.906″	E 31° 36′ 26.695″	18.7	8.7	368	0.0035	0.0123	0.0007	0.0001	0.0024	0.0035	0.0093	ND	0.046
7	N 30° 44′ 33.264″	E 31° 36′ 6.414″	19	8.5	359	0.0055	0.0033	0.0013	0.0002	0.0015	0.0015	ND	0.0007	0.1068
8	N 30° 43′ 47.110″	E 31° 36^′^ 4.014″	20.1	8.6	370	0.0049	0.0049	0.002	0.0002	0.0001	0.0083	0.0109	0.001	0.2674
9	N 30° 41^′^ 11.46″	E 31° 35^′^ 10.39″	19.5	8.6	373	0.0056	0.0013	0.0033	ND	ND	0.003	ND	0.0001	ND
10	N 30° 40^′^ 10.56″	E 31° 34^′^ 43.2″	20.3	8.6	372	0.0023	0.0013	0.0021	0.0001	0.0003	0.0033	0.011	0.0002	0.2339
11	N 30° 37^′^ 33.6″	E 31° 32^′^ 17.3″	20.2	8.5	375	0.0043	0.0025	0.0018	0.0002	0.0058	0.0024	0.01	ND	0.1385
12	N 30° 36^′^ 19.8″	E 31° 31^′^ 8.89″	20	8.5	376	0.0028	0.0239	0.0007	0.0001	0.0028	0.0048	0.0021	ND	0.043
13	N 30° 35^′^ 10.6″	E 31° 28^′^ 13.1″	20.4	8.4	379	0.0071	0.0026	0.0006	ND	0.0095	0.0048	0.0063	ND	0.1463
14	N 30° 35^′^ 36.4″	E 31° 26^′^ 41.4″	20.3	8.6	380	0.0065	0.0033	0.0014	0.0001	0.0051	0.005	0.0019	0.001	0.129
15	N 30° 34^′^ 36.86″	E 31° 24^′^ 56.3″	19.6	8.6	378	0.0019	0.0031	0.0004	0.0002	0.0108	0.0074	0.0008	ND	0.1337
16	N 30° 33^′^ 20.83″	E 31° 23^′^ 9.27″	19.8	9	374	0.0041	0.0016	0.0003	0.0001	0.0022	0.0017	0.002	0.0005	0.0428
17	N 30° 30^′^ 38.9″	E 31° 19^′^ 46.86″	19.7	8.6	383	0.004	0.0184	0.0006	0.0002	0.0029	0.0059	0.0018	ND	0.1176
18	N 30° 29^′^ 48.617″	E 31° 19^′^ 25.436″	19.7	8.6	382	0.0051	0.009	0.0019	0.0003	0.0018	0.0059	0.0036	ND	0.1678
19	N 30° 29^′^ 4.259″	E 31° 16^′^ 7.528″	19.8	8.6	378	0.0074	0.0042	0.0021	0.0002	0.0044	0.0054	0.006	ND	0.1558

**Table 3:** Water physiochemical properties and heavy metals concentrations (ppm) of water samples of Bahr Mouse in the summer season 2019

Station	Latitude	Longitude	T (°C)	pH	TDS (mg/L)	EC (μS)	Fe	Mn	Cu	Cd	Pb	Zn	Ni	Co	Cr
1	N 30° 55^′^ 16.697″	E 31° 41^′^ 35.123″	29.6	8.2	195	391	0.0709	0.0018	0.0025	ND	0.0037	ND	0.0082	0.0132	0.0657
2	N 30° 52^′^ 56.914″	E 31° 38^′^ 32.474″	31	8.3	190	382	0.0535	0.0010	0.0005	0.0001	ND	ND	0.0193	0.0258	0.0232
3	N 30° 50^′^ 47.302″	E 31° 36^′^ 52.077″	30	8.4	191	382	0.0568	0.0009	0.0010	ND	ND	0.0004	0.0066	0.0303	0.0233
4	N 30° 49^′^ 54.102″	E 31° 37^′^ 11.965″	30	8.4	191	383	0.0581	ND	0.0002	0.0001	0.0560	ND	ND	0.0106	0.0028
5	N 30° 48^′^ 9.805″	E 31° 37^′^ 48.181″	30.3	8.5	182	365	0.0988	0.0006	0.0001	ND	ND	ND	0.0017	0.0023	0.0482
6	N 30° 45^′^ 47.906″	E 31° 36^′^ 26.695″	29.8	8.5	192	387	0.1049	0.0015	ND	ND	ND	0.0007	ND	0.0105	0.0287
7	N 30° 44^′^ 33.264″	E 31° 36^′^ 6.414″	30.4	8.5	189	378	0.0096	0.0014	0.0022	0.0001	ND	0.0019	0.0175	0.0108	0.0132
8	N 30° 43^′^ 47.110″	E 31° 36^′^ 4.014″	30.6	8.7	184	368	0.0338	0.0016	0.0026	ND	ND	0.0010	0.0093	0.0121	0.0033
9	N 30° 41^′^ 11.46″	E 31° 35^′^ 10.39″	30	8.6	193	389	0.0159	ND	0.0034	ND	0.0007	ND	0.0169	0.0161	ND
10	N 30° 40^′^ 10.56″	E 31° 34^′^ 43.2″	31.8	8.6	193	387	0.0369	0.0006	0.0012	ND	ND	ND	0.0101	0.0102	0.0323
11	N 30° 37^′^ 33.6″	E 31° 32^′^ 17.3″	33	8.6	186	371	0.0627	0.0003	0.0008	ND	ND	0.0002	0.0092	0.0258	0.0168
12	N 30° 36^′^ 19.8″	E 31° 31^′^ 8.89″	33.3	8.6	190	380	0.0106	0.0005	0.0014	0.0001	ND	ND	0.0156	0.0026	0.0241
13	N 30° 35^′^ 10.6″	E 31° 28^′^ 13.1″	32	8.7	190	383	0.0276	ND	0.0014	0.0003	ND	ND	0.0092	ND	0.0152
14	N 30° 35^′^ 36.4″	E 31° 26^′^ 41.4″	34.5	8.7	188	375	0.0578	0.0015	0.0008	ND	ND	0.0010	0.0123	0.0145	0.0484
15	N 30° 34^′^ 36.86″	E 31° 24^′^ 56.3″	32.5	8.7	177	352	0.0060	0.0006	0.0017	ND	ND	0.0004	0.0128	0.0217	0.0229
16	N 30° 33^′^ 20.83″	E 31° 23^′^ 9.27″	33	8.7	182	364	0.0639	0.0022	0.0009	0.0008	ND	ND	0.0125	0.0044	0.0102
17	N 30° 30^′^ 38.9″	E 31° 19^′^ 46.86″	33	8.7	184	368	0.0725	0.0024	0.0005	0.0009	ND	0.0009	0.0124	0.0543	0.0479
18	N 30° 29^′^ 48.617″	E 31° 19^′^ 25.436″	32.7	8.7	185	370	0.1006	0.0008	ND	0.0002	ND	ND	0.0180	0.0047	0.0182
19	N 30° 29^′^ 4.259″	E 31° 16^′^ 7.528″	33	8.8	177	355	0.0212	0.0009	0.0003	0.0005	ND	ND	0.0138	0.0083	0.0214

**Table 4:** Comparison of heavy metal concentrations (ppm) in surface water samples from the Bahr Mouse with the international permitted levels

Heavy metal	Present work	Drinking water standards			Irrigation standards
	Mean	EWQS (2007)	WHO (2022)	USEPA (2018)	FAO (1994)
Fe	0.0275	0.3	0.3	0.3	< 5
Mn	0.0041	0.1	0.4	0.05	0.2
Cu	0.0013	2	2	1.3	< 0.2
Cd	0.0002	0.003	0.003	0.005	< 0.01
Pb	0.0054	0.01	0.01	0.015	< 5
Zn	0.0036	3	3	5	< 2
Ni	0.0089	0.02	0.07	0.1	< 0.2
Co	0.0116	2	0.05	0.1	< 0.05
Cr	0.0827	0.05	0.05	0.1	< 0.1

**Table 5:** Comparison of heavy metal concentrations (ppm) in surface water samples from the Bahr Mouse with other locations

Location	Fe	Mn	Cu	Cd	Pb	Zn	Ni	Co	Cr	References
Bahr Mouse water	0.0275	0.0041	0.0013	0.0002	0.0054	0.0036	0.0089	0.0116	0.0827	Present Work
Bahr Mouse water	nd	0.658 ± 0.087	1.981 ± 0.859	1.031 ± 0.067	4.148 ± 1.251	10.194 ± 2.040	0.328 ± 0.066	6.275 ± 3.425	2.378 ± 1.610	El-Sayed et al. (2011)
Bahr Mouse water	0.055	0.03	0.006	0.12	0.129	0.085	0.136	0.017	nd	Abdel-Hamed et al. (2018)
Damietta Nile Branch, Egypt	0.221	0.06	0.012	0.002	0.02	0.191	0.011	nd	0.1	El-Bady (2019)
Degirmendere Dam, Turkey	0.0433	0.0046	0.0058	0.0007	0.0011	0.0308	0.0030	nd	0.0012	Ustaoglu (2020)
Major lakes, Kenya	nd	0.0589	nd	0.0005	0.0047	0.0078	0.0162	0.00078	0.0027	Githaiga et al. (2021)

Furthermore, Pearson’s correlation coefficient analysis illustrated a moderately significant positive correlation between Fe content and Cr (*r* = 0.523), along with a significant negative correlation coefficient with Cu (*r* =  − 0.548) at the 0.01 level (Table 6). This elucidates the interplay between these metals and their dynamics within aquatic environments. Manganese (Mn), a naturally occurring metal vital for human and animal health (Hem, 1985; Kacmaz, 2020), exhibited concentrations ranging from 0.0011 to 0.0239 ppm during winter and non-detectable to 0.0024 ppm during summer. These concentrations remained below the established limit of 0.05 mg/L for Secondary Drinking Water Regulation (SDWR), which is not specifically regulated by the USEPA. However, they are notably comparable to previous studies by Abdel-Hamed et al. (2018) on Bahr Mouse water (0.03 ppm). Correlation analysis indicated a moderately significant positive relationship between Mn and Cu (*r* = 0.655) at the 0.01 level, as well as a low significant negative correlation coefficient with Cd (*r* =  − 0.443) at the 0.05 level (Table 6).

**Table 6:** Correlation analysis between the physiochemical parameters of water and the concentrations of heavy metals in the study area

Variables	Temperature	PH	TDS (mg/L)	EC μS	Fe	Mn	Cu	Cd	Pb	Zn	Ni	Co	Cr
Temperature	1												
PH	0.484*	1											
TDS (mg/L)	− 0.209	–0.665**	1										
EC μS	–0.458*	–0.593**	0.499*	1									
Fe	− 0.168	− 0.004	0.078	0.075	1								
Mn	− 0.282	− 0.056	0.336	0.304*	− 0.023	1							
Cu	− 0.236	− 0.136	0.156	0.344*	–0.548**	0.655**	1						
Cd	0.077	0.016	− 0.229	–0.427*	0.011	–0.443*	− 0.163	1					
Pb	− 0.130	0.027	0.100	0.063	− 0.021	− 0.179	− 0.215	− 0.006	1				
Zn	− 0.051	− 0.104	0.050	0.333*	− 0.097	0.162	0.142	–0.350*	− 0.183	1			
Ni	0.500**	0.138	− 0.070	–0.334*	− 0.148	− 0.170	− 0.050	− 0.091	− 0.242	− 0.155	1		
Co	0.043	− 0.027	− 0.225	− 0.142	− 0.005	− 0.189	− 0.002	0.051	− 0.239	0.374*	− 0.221	1	
Cr	− 0.078	− 0.391	0.444*	0.133	0.523**	0.227	− 0.187	0.112	–0.306*	− 0.038	− 0.130	0.191	1

^**^Correlation is significant at the 0.01 level (2-tailed)

^*^Correlation is significant at the 0.05 level (2-tailed)

Copper (Cu) concentration in the studied water samples showed that the highest recorded values of 0.0033 and 0.0034 ppm were observed at station 9 (Hehia) during winter and summer, respectively. Conversely, the lowest Cu values (0.0003 ppm) during winter were noted at station 16 (Kafr Ar Rubaemayah, Minya Al Qamh) and non-detectable during summer at station 6 (Al Ibrahimiya) and station 18 (Near Kafr El Saedy, Minya Al Qamh). The average Cu concentration across all samples was 0.0013 ppm (Fig. 4). As such, Cu concentrations remain within the safety limits outlined by EWQS (2007) and below the recommended thresholds set by WHO (2022), USEPA (2018), and FAO (1994) and they are low when compared with other local and international studies (Table 5).

The concentration of cadmium (Cd) remained below the detection limit in most of the water samples, and lower than the recommended limits outlined by Egyptian Law, as well as guidelines established by WHO (2022), USEPA (2018), and FAO (1994) (Table 4). The highest measured Cd concentration was 0.0003 ppm during winter at station 18 (Near Kafr El Saedy, Minya Al Qamh) and 0.0009 ppm during summer at station 17 (Kafr Badawi, Minya Al Qamh). These values were notably low in comparison with other pertinent local and international studies (Table 5). Lead (Pb) concentration showed seasonal fluctuations, with the highest value (0.0108 ppm) during winter at station 15 (Zagazig) and 0.0560 ppm during summer at station 4 (Kafr Saqr). Conversely, the lowest Pb concentration fell below the detection limit during winter at station 9 (Hehia) and in most collected water samples during summer (Table 3). The average Pb concentration of 0.0054 ppm (Fig. 4) remained below the safety limits stipulated by USEPA (2018) and WHO (2011) as shown in Table 4. Additionally, these concentrations were lower when juxtaposed with data from various local and international studies (Table 5).

Zinc (Zn), crucial for numerous biological processes (Kacmaz, 2020), exhibited concentrations ranging from 0.0011 to 0.0083 ppm during winter, and non-detectable to 0.0019 ppm during summer. These concentrations are below the established thresholds (Table 4) and are lower than those observed in comparative studies (Table 5). Zn content has a low significant positive correlation with Co (*r* = 0.374) at the 0.05 level (Table 6). Industrial effluents and wastewater are potential sources of zinc oxide, used as an oxidizing agent to create the fatty acids utilized in soap production (El-Sayed et al., 2011).

Nickel (Ni) concentration varied with the highest values recorded at stations 10 (Hehia) and 8 (Kofour Negm Village, Al Ibrahimiya) of 0.0110 and 0.0109 ppm, respectively, while reaching 0.0193 ppm during summer at station 2 (Hanout, Kafr Saqr). Conversely, Ni contents were non-detectable at various stations during both winter and summer. The average Ni concentration was 0.0089 ppm (Fig. 4), all within established safe limits (Table 4) and comparatively lower than data from local and international studies (Table 5).

Cobalt (Co), essential as a trace mineral for various organisms including humans and animals, displayed variable concentrations across seasons. Co contents ranged from 0.0026 ppm at station 1 (Awlad Saqr) to 0.0001 ppm in station 9 (Hehia) during winter, and from 0.0543 ppm at station 17 (Minya Al Qamh) to 0.0023 ppm at station 5 (Kafr Saqr) during summer. These concentrations remained within the safety limits outlined by EWQS (2007) and below the recommended limits of WHO (2022), USEPA (2018), and FAO (1994) (Table 4). Additionally, Co levels in most samples were lower than the mean concentrations reported in pertinent studies (Abdel-Hamed et al., 2018; El-Sayed et al., 2011) (Table 5).

The distribution of heavy metals in the studied samples from the Bahr Mouse stream confirms the results by Abd El-Wahed (2007), who indicated that Fe, Mn, Zn, and Pb concentrations in surface water were below the standard permissible limits. On the other hand, El-Sayed (2011) reported that heavy metals have high concentrations, especially zinc and chromium. This may be attributed to wastes of industrial activities and wastewater. The levels of metals exhibited seasonal fluctuations, where all heavy metal concentrations in water samples (except Cu) showed significant variations between seasons (P-values < 0.05). Summer has the highest concentrations of Fe, Cu, Cd, Pb, Ni, and Co (0.1049, 0.0034, 0.0009, 0.056, 0.0193, and 0.0543 ppm), respectively. In contrast, the highest concentrations of Mn, Zn, and Cr were recorded in winter (0.0239, 0.0083, and 0.4518 ppm) respectively. Abd El-Aal (2020) ascribed the rise in metal concentration in water during hot seasons to the release of heavy metals from sediment to the water under the influence of both high temperature and fermentation process which is caused by the organic materials breakdown. Numerous research studies (i.e., El-Safy & Al-Ghannam, 1996; Hamed, 1998) carried out on the seasonal changes of heavy metal contents. They suggested that these seasonal variations could be a result of industrial activity and wastewater.

Correlation analysis revealed a moderately significant positive correlation between temperature values and Ni (*r* = 0.5) at 0.01 level. Additionally, TDS exhibited a low significant positive correlation with Cr (*r* = 0.444) at 0.05 level (Table 6). Moreover, EC values have a low significant positive correlation with Mn, Cu, and Zn (*r* = 0.304, 0.344, and 0.333, respectively) at the 0.05 level, while demonstrating a low significant negative correlation with Cd and Ni (*r* =  − 0.427 and 0. − 334, respectively) at the same level (Table 6).

### Heavy metal contamination and water quality assessment

**Fig. 5:**
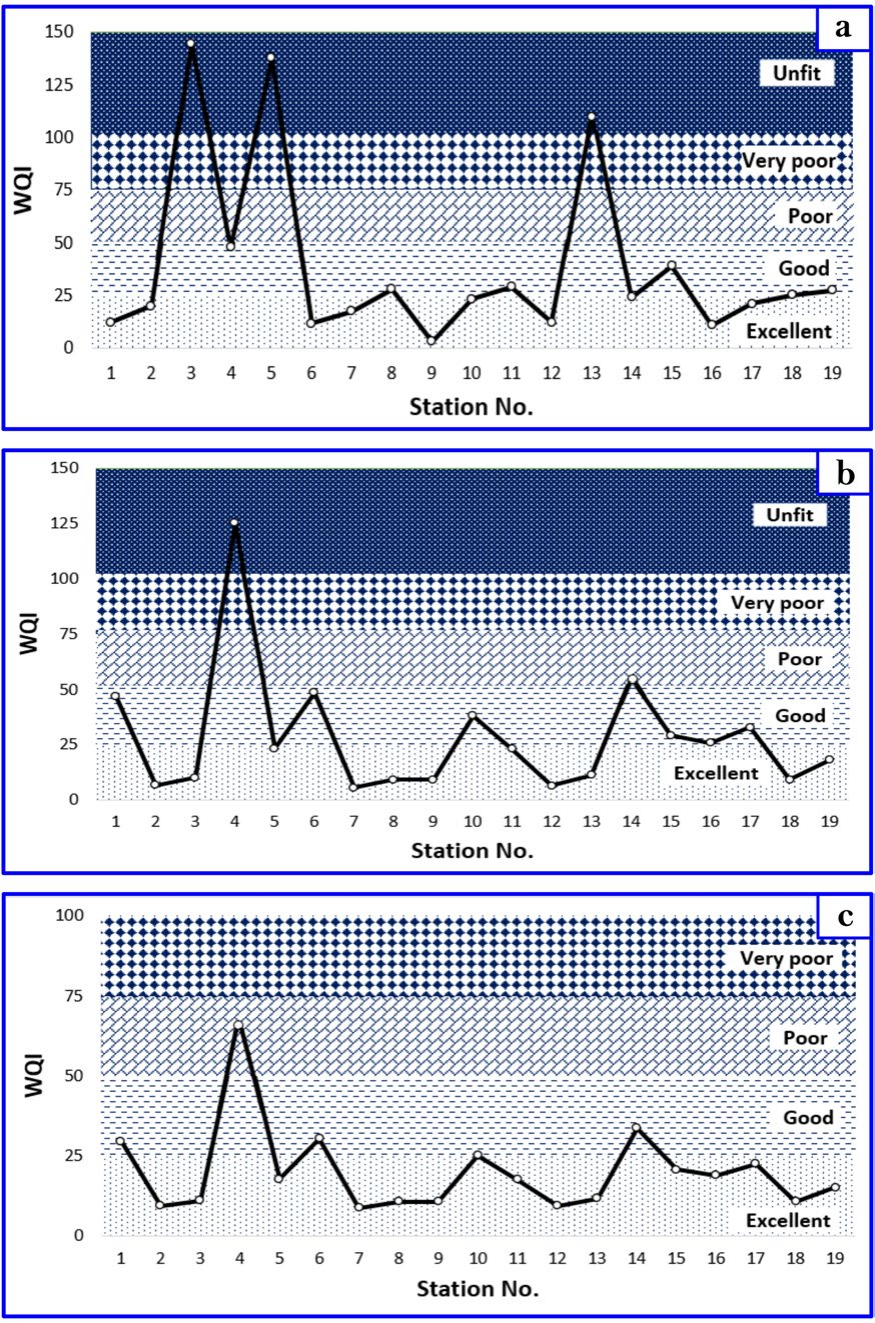
WQI of surface water of Bahr Mouse stream (East Nile Delta, Egypt) in winter season (**a**), in summer season (**b**), and during the study period (**c**)

The assessment of Bahr Mouse surface water quality was conducted utilizing the Weighted Arithmetic Water Quality Index (WAWQI). This method amalgamates various physicochemical parameters through mathematical equations to generate a single value representative of the overall quality status. The selection of ten parameters for calculating the Water Quality Index (WQI) for potable water encompasses pH, total dissolved solids (TDS), iron (Fe), manganese (Mn), copper (Cu), cadmium (Cd), lead (Pb), zinc (Zn), nickel (Ni), and chromium (Cr). The determination of WQI values adhered to the water quality standards outlined by the World Health Organization and the Egyptian Ministry of Health. The WQI values ranged from 144.28 (indicating an unfit condition) at Station 3 in Kafr Saqr City to 2.92 (representing an excellent condition) at Station 9 in Hehia City during winter (Fig. 5a).

Similarly, during summer, the WQI values spanned from 125.3 (indicative of an unfit condition) at Station 4 in Kafr Saqr City to 5.39 (demonstrating an excellent condition) at Station 7 in Al-Ibrahimiya City (Fig. 5b). Analysis indicated that approximately 52.6% of water stations had WQI values below 25, signifying excellent water quality, while 31.6% of stations registered WQI values ranging between 25 and 50, indicating good water quality for drinking during winter (Table 7). However, three samples (3, 5, and 13) with elevated WQI (> 100) denoted unsuitable water quality for both drinking and fish culture (Fig. 5a). Of these, samples 3 and 5 were sourced from Kafr Saqr City, characterized by agricultural activities, urban development, and significant household wastes discharged into water bodies. Sample 13 originated from Zagazig City, where urban activities and industrial operations, such as the red brick factory, contributed to water pollution.

**Table 7:** Summary of WQI, HEI, Cd, HMPI, and PI of Bahr Mouse stream, Sharkiya Governorate, East Nile Delta, Egypt

Station	Winter					Summer					Mean				
	WQI	HEI	Cd	HMPI	PI	WQI	HEI	Cd	HMPI	PI	WQI	HEI	Cd	HMPI	PI
1	12.12	1.604	− 6.396	12.10	0.200	46.79	2.043	− 3.957	46.76	0.341	29.46	1.823	− 5.177	29.43	0.271
2	19.85	1.131	− 6.869	19.83	0.141	6.69	0.954	− 5.046	6.67	0.159	9.41	1.043	− 5.957	13.25	0.150
3	144.28	9.377	2.377	144.32	1.172	9.94	0.753	− 4.248	27.60	0.125	11.03	5.065	− 0.935	85.96	0.649
4	47.67	0.780	− 6.220	47.61	0.110	125.30	5.883	0.883	125.31	1.177	68.71	3.332	− 2.668	86.46	0.643
5	137.74	6.056	− 0.944	137.76	0.757	23.24	1.189	− 2.981	23.16	0.238	17.68	3.623	− 1.962	80.46	0.497
6	11.73	1.370	− 6.630	11.71	0.171	48.59	0.928	− 3.072	48.36	0.232	30.36	1.149	− 4.851	30.04	0.202
7	17.42	2.380	− 4.620	17.40	0.298	5.39	0.585	− 6.415	5.36	0.084	8.76	1.482	− 5.518	11.38	0.191
8	27.96	5.613	− 2.387	27.94	0.702	9.10	0.317	− 5.683	8.80	0.053	10.61	2.965	− 4.035	18.37	0.377
9	2.92	0.025	− 3.975	0.34	0.003	9.09	0.353	− 2.875	9.00	0.092	10.61	0.189	− 3.425	4.67	0.047
10	23.20	4.912	− 3.088	23.18	0.029	38.10	0.915	− 4.085	37.90	0.183	25.11	2.913	− 3.587	30.54	0.106
11	29.06	3.582	− 4.418	29.04	0.448	22.94	0.678	− 5.322	22.70	0.113	17.53	2.130	− 4.870	25.87	0.280
12	12.02	1.274	− 6.726	12.00	0.159	6.46	0.775	− 5.225	6.43	0.129	9.29	1.025	− 5.975	9.22	0.144
13	109.88	3.998	− 3.002	109.90	0.500	11.23	0.628	− 4.372	11.20	0.126	11.68	2.313	− 3.687	60.55	0.313
14	24.10	3.183	− 4.817	24.08	0.398	55.16	1.341	− 4.659	55.00	0.223	33.64	2.262	− 4.738	39.54	0.311
15	38.79	3.849	− 4.151	38.78	0.481	29.16	0.663	− 5.337	28.92	0.111	20.64	2.256	− 4.744	33.85	0.296
16	10.72	1.156	− 6.844	10.69	0.145	25.76	0.868	− 5.132	25.74	0.145	18.94	1.012	− 5.988	18.21	0.145
17	20.85	2.796	− 5.204	20.83	0.349	32.76	1.683	− 5.317	32.73	0.240	22.44	2.240	− 5.260	26.78	0.295
18	25.17	3.730	− 4.270	25.15	0.466	9.22	1.025	− 3.975	9.18	0.205	10.67	2.377	− 4.123	17.17	0.336
19	27.40	3.746	− 4.254	27.38	0.468	18.00	0.865	− 5.135	17.96	0.144	15.06	2.306	− 4.694	22.67	0.306
Mean	39.10	3.19	− 4.34	38.95	0.37	28.05	1.18	− 4.31	28.88	0.22	20.94	2.17	− 4.32	35.56	0.29

During summer, approximately 58% of water stations had WQI values below 25, indicating excellent water quality for drinking, while 31.6% of stations recorded WQI values ranging between 25 and 50, representing good water quality except for two samples (4 and 14) with elevated WQI values, signifying poor and unfit water quality for drinking, respectively (Fig. 5b; Table 7). The former sample was sourced from Kafr Saqr City, where agricultural activities and household waste discharge were prominent, while the latter was from Zagazig City, characterized by urban activities and industrial effluents, including those from adjacent red brick factories.

The mean WQI values across different stations depicted in Fig. 5c and Table 7 showed that 73.7% of water stations had WQI values below 25, representing excellent water quality, while 21.1% had WQI values ranging between 25 and 50, indicating good water quality (Fig. 5c). Notably, only station 4 (located in Kafr Saqr City) had WQI value between 50 and 75, revealing poor water quality which is unsuitable for drinking, attributed to the discharge of household wastes into the water stream by local inhabitants. The overall mean WQI values for all stations during different seasons were 39.1 and 28.05 (during winter and summer, respectively), indicating a good condition (Table 7). On average, the water quality of Bahr Mouse was deemed good (20.94) and suitable for drinking. The observed ranges of WQI values greater than 100 as previously mentioned, underscores the spatial and temporal variability in water quality across the studied region.

The Fe content exhibits a moderately significant positive correlation with Cr, as well as Mn with Cu, implying similar geochemical behavior of these metals (Gu et al., 2016). In addition, Zn content has a low significant positive correlation with Co, indicating potential leaching from anthropogenic sources such as agricultural and domestic waste (Nour & Nouh, 2020; Prasanna et al., 2012).

**Fig. 6:**
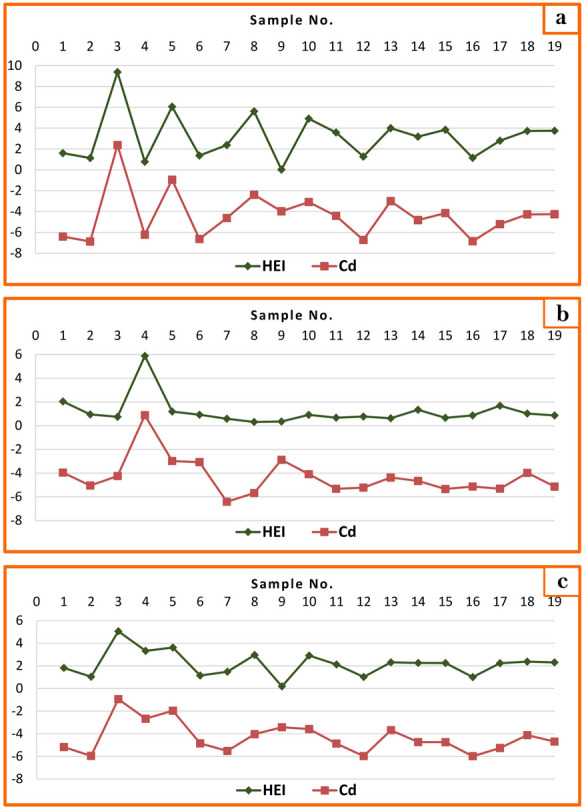
Graphical representation of HEI and Cd of surface water Bahr Mouse stream (East Nile Delta, Egypt) in winter season (**a**), in summer season (**b**), and during the study period (**c**)

Analysis presented in Table 7, Fig. 6a, and Fig. 6b reveals higher values of the Heavy Metal Pollution Index (HEI) during winter compared to summer, suggesting rainfall acted as a driver of water pollution. Station 3 (9.38) during winter and station 4 (5.88) during summer exhibited the maximum HEI levels in Bahr Mouse surface water (Table 7). The average HEI for winter and summer samples was 3.19 and 1.18 respectively. Mean HEI values across different stations ranged from 0.189 to 5.065 (Fig. 6c), indicating low-contamination levels (< 10), and suitability of the water for drinking purposes.

Regarding contamination degree (Cd) during winter, all stations except station 3 (2.37) in Kafr Saqr City were classified as low-contaminated (Fig. 6a; Table 7). In summer, all stations remained low-contaminated (Fig. 6b). The average contamination degree average for all stations during different seasons was − 4.34 in winter and − 4.31 in summer, classified as low-contaminated (Table 7).

**Fig. 7:**
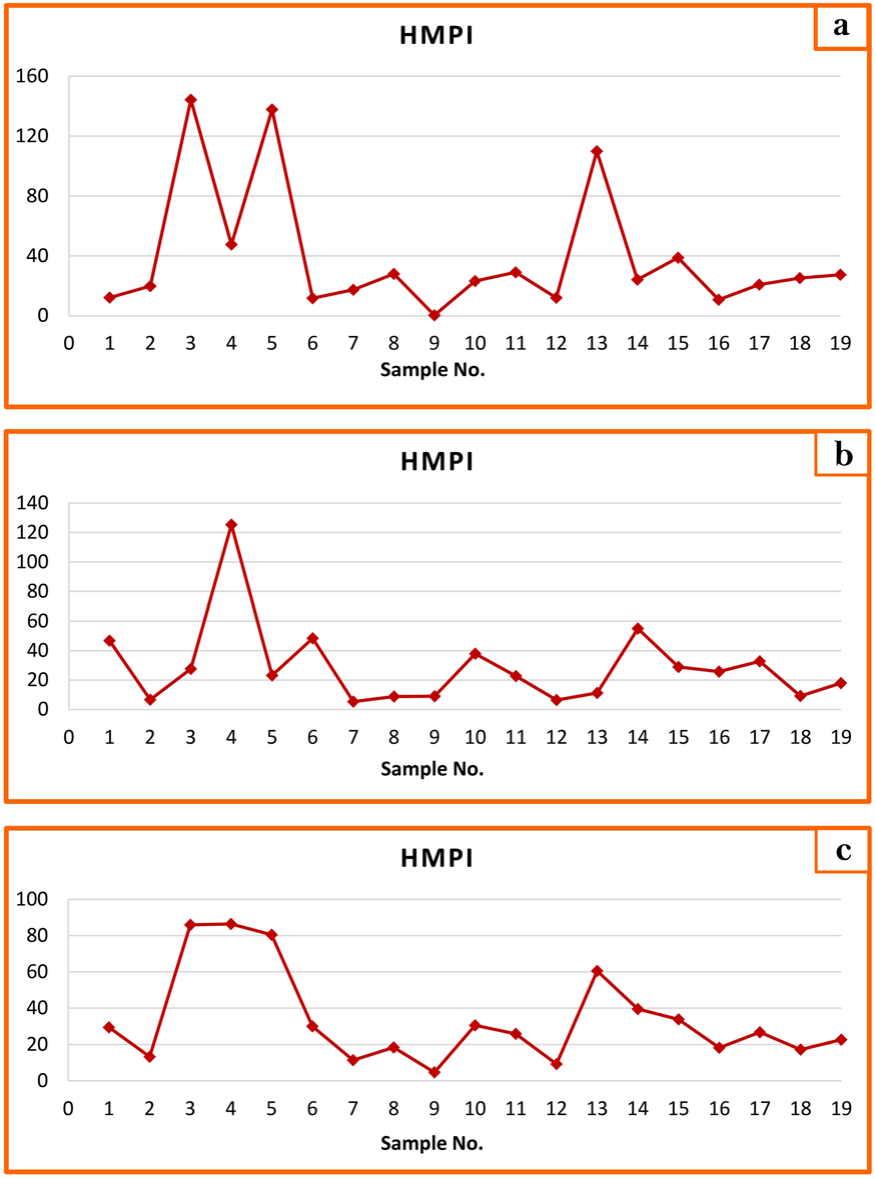
Graphical representation of HMPI of surface water of Bahr Mouse stream (East Nile Delta, Egypt) in winter season (**a**), in summer season (**b**), and during the study period (**c**)

During winter, most stations had low Heavy Metal Pollution Index (HMPI) values below the critical value of 100, except stations 3, 5 (Kafr Saqr City), and 13 (Zagazig City) which had 144.32, 137.76, and 109.9 respectively (Fig. 7a; Table 7). In summer, all stations had low HMPI values except station 4 in Saqr City, which exceeded the critical value (125.31) (Fig. 7b). Mean HMPI values across stations Fig. 7c and Table 7, showed 100% of water stations with HMPI < 100, denoting safe water for drinking. The overall mean HMPI values for all stations during different seasons were 38.95 in winter and 28.88 in summer (Table 7), suggesting Bahr Mouse water was generally safe (35.56) and suitable for drinking.

**Fig. 8:**
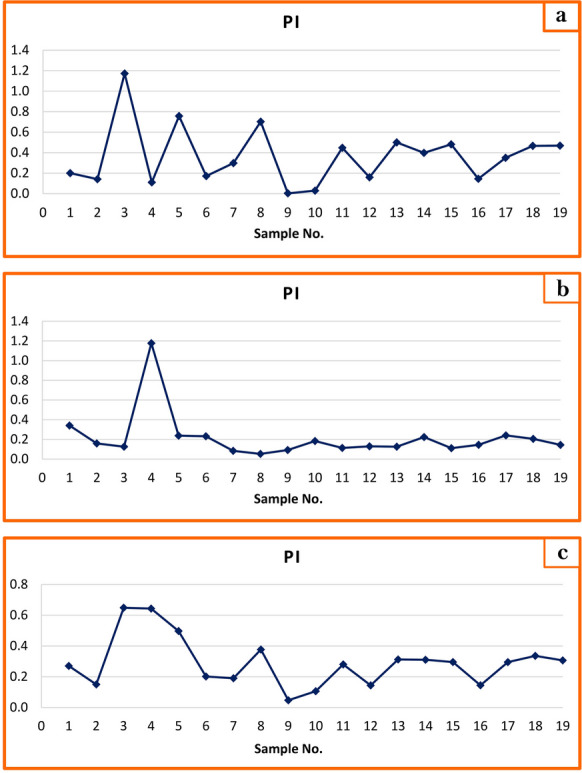
Graphical representation of PI of surface water of Bahr Mouse stream (East Nile Delta, Egypt) in winter season (**a**), in summer season (**b**), and during the study period (**c**)

Similarly, Pollution Index (PI) values during both winter and summer seasons, classified all stations as unaffected (< 1) by heavy metals except station 3 (Kafr Saqr City) in winter and station 4 (Kafr Saqr City) in summer which were slightly affected (Figs. 8a and b). Average PI values confirmed no significant impact of heavy metal across sites and their water was suitable for drinking (Fig. 8c). Collectively, graphical representations of the five water quality indices (WQI, HEI, Cd, HMPI, and PI) demonstrate consistency in results, affirming Bahr Mouse water’s suitability for drinking and irrigation purposes.

## Conclusions

Based on the comprehensive analysis of heavy metal contamination and environmental risk assessment conducted in the Bahr Mouse stream, East Nile Delta, Egypt, several key findings emerge as follows:

- Physiochemical parameter analysis revealed seasonal variations in water temperature, pH, electrical conductivity (EC), and total dissolved solids (TDS), with temperatures exceeding permissible limits during summer, while pH, EC, and TDS remained within acceptable ranges outlined by international standards. These variations underscore the importance of monitoring these parameters for assessing water quality and potential environmental risks.
- Heavy metal analyses demonstrated the presence of various metals in the surface water samples, with concentrations fluctuating across seasons. Notably, most heavy metal concentrations remained within safe limits established for drinking water, although seasonal variations and spatial distribution patterns were observed. Correlation analyses provided insights into the interplay between different heavy metal species and their environmental dynamics.
- Water quality assessment utilizing the Weighted Arithmetic Water Quality Index (WAWQI) highlighted overall good water quality in the Bahr Mouse stream, with some localized instances of poor water quality attributed to anthropogenic activities such as agricultural runoff, urban development, and industrial effluents. The assessment also revealed seasonal variations in water quality, with slightly high contamination levels observed during winter months.
- Additionally, the Heavy Metal Pollution Index (HEI), contamination degree (Cd), Heavy Metal Pollution Index (HMPI), and Pollution Index (PI) methodologies corroborated the findings of the WQI assessment, indicating consistency in evaluating water quality across different indices. These indices collectively affirmed the suitability of Bahr Mouse water for drinking and irrigation purposes, despite localized contamination hotspots.

In summary, the study provides valuable insights into the heavy metal contamination and environmental risks associated with surface water in the Bahr Mouse stream, offering essential information for policy makers, environmental regulators, and stakeholders to develop effective mitigation strategies and safeguard water resources in the East Nile Delta region of Egypt. Ongoing monitoring and management efforts are crucial to maintaining water quality standards and ensuring the sustainability of freshwater ecosystems in the area.
